# *DeepShape*: estimating isoform-level ribosome abundance and distribution with Ribo-seq data

**DOI:** 10.1186/s12859-019-3244-0

**Published:** 2019-12-20

**Authors:** Hongfei Cui, Hailin Hu, Jianyang Zeng, Ting Chen

**Affiliations:** 1 Institute for Artificial Intelligence and Department of Computer Science and Technology, Tsinghua University, Beijing, China; 2 DonLinks School of Economics and Management, University of Science and Technology Beijing, Beijing, China; 3 School of Medicine, Tsinghua University, Beijing, China; 4 Institute for Interdisciplinary Information Sciences, Tsinghua University, Beijing, China

**Keywords:** Ribo-seq, Translation, Transcript-level, Multiple alignment

## Abstract

**Background:**

Ribosome profiling brings insight to the process of translation. A basic step in profile construction at transcript level is to map Ribo-seq data to transcripts, and then assign a huge number of multiple-mapped reads to similar isoforms. Existing methods either discard the multiple mapped-reads, or allocate them randomly, or assign them proportionally according to transcript abundance estimated from RNA-seq data.

**Results:**

Here we present ***DeepShape***, an RNA-seq free computational method to estimate ribosome abundance of isoforms, and simultaneously compute their ribosome profiles using a deep learning model. Our simulation results demonstrate that ***DeepShape*** can provide more accurate estimations on both ribosome abundance and profiles when compared to state-of-the-art methods. We applied ***DeepShape*** to a set of Ribo-seq data from PC3 human prostate cancer cells with and without PP242 treatment. In the four cell invasion/metastasis genes that are translationally regulated by PP242 treatment, different isoforms show very different characteristics of translational efficiency and regulation patterns. Transcript level ribosome distributions were analyzed by “Codon Residence Index (CRI)” proposed in this study to investigate the relative speed that a ribosome moves on a codon compared to its synonymous codons. We observe consistent CRI patterns in PC3 cells. We found that the translation of several codons could be regulated by PP242 treatment.

**Conclusion:**

In summary, we demonstrate that ***DeepShape*** can serve as a powerful tool for Ribo-seq data analysis.

## Background

Regulation of mRNA translation is critical to the coordinated and controlled expression of many important biological pathways, which have great impact on human health states [1, 2]. The ribosome, a complex molecular machine, plays a central role in the translation process [3]. A ribosome binding to a transcript implies the synthesis of a new peptide. Multiple factors can influence ribosome binding and also ribosome distribution at transcripts, including composition of nucleotides along its current, upstream, and downstream RNA sequences [4, 5]; instantaneous environmental stress [6, 7]; and state of the cell [8]. Therefore, distributions of ribosome binding in a transcriptome reflect underlying translation process, and thus is strongly related to phenotype state.

Ribosome sequencing (Ribo-seq) is an important approach to obtain ribosome distributions by sequencing “ribosome-protected fragments” (RPFs) [9]. Computational analysis involves mapping sequencing Ribo-seq reads into transcripts and counting number of reads at each position to obtain distributions of ribosomes along the whole transcriptome. The sites where Ribo-seq reads are piled up suggest ribosome stalling or translation slow-down, which could be caused by factors such as codon usage bias [10], codon co-occurrence bias [5] and proline codons [11]. The stalling events can be related to translation regulation [12, 13]. Besides ribosome stalling, ribosome profiles also reveal unexpected translation events on non-coding regions [14]. Overall, ribosome profiling brings insight to the process and regulation of translation.

**Fig. 1:**
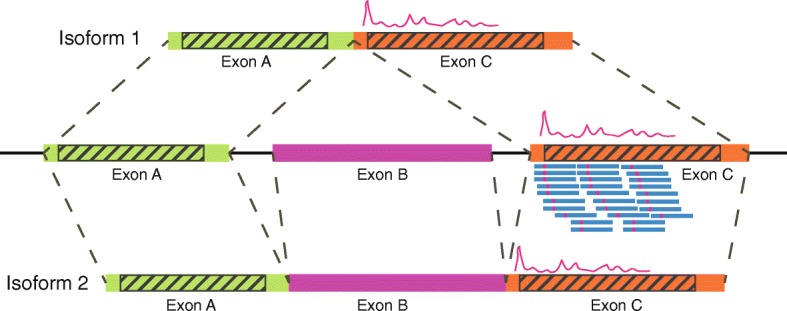
An example of ribosome distribution calculation on two isoforms. There are two isoforms from a gene. With existing methods that allocate multiple-mapped reads proportionally to each transcript, the central part (where reads are completely within the exon, not on the junction) of an exon in two isoforms will have the same ribosome distributions, regardless of ribosome context (e.g., the shaded area in Exon C)

Computational tools, or pipelines, have been developed for the analysis of Ribo-seq data, including Ribomap [15], RiboProfiling [16], RiboSeqR [17], Plastid [18] and Ribogalaxy [19]. However, most of these tools were targeted at gene-level ribosome analysis, instead of more informative transcript-level analysis. The challenge is that Ribo-seq reads (~ 30 bp) are too short to be mapped accurately to multiple alternatively spliced transcripts, resulting in a large proportion of multiple mapped reads [15]. This has become the main barrier against obtaining precise ribosome profiling at transcript level. A simple strategy is to discard these multiple-mapped reads. However, as Wang et al. show in their study [15], there may be less than 10% of all reads can be mapped to unique positions on transcript sequences. Discarding all multiple mapped reads would lose major information in this process. Another strategy is to allocate multiple-mapped reads randomly, as adopted by RiboSeqR [17], or the “*star prime*” method evaluated in the work of Wang et al. [15], but this may introduce bias to transcripts of low-abundance genes, and in addition, it has been shown that this strategy does not perform well on transcript level analysis [15]. A more robust strategy, adopted by Ribomap [15], is to take advantage of RNA-seq data generated simultaneously with Ribo-seq and allocate multiple-mapped reads to transcripts according to the transcript abundance estimated from the RNA-seq data. However, this would involve two assumptions: (1) translation activity is coherent to the abundance of mRNA molecules, and (2) that ribosome profiles at regions shared by two different isoforms of a gene are exactly the same. Strictly speaking, for the first assumption, mRNA abundance is not equal to the translating mRNA abundance because some of the mRNAs may be unoccupied by any ribosomes, which is linked to different levels of initiation efficiency [9, 20], and neither is translating mRNA abundance equal to ribosome abundance on them because different translating mRNAs have different elongation velocities which lead to different ribosome densities on them [20–22]. For the second assumption, as many studies have shown, the upstream and downstream sequence compositions are important and can affect how fast ribosomes move along a transcript [4, 5]. If a frame shift exists between two isoforms because of different upstream exons, their ribosome profiles are most likely to be different. Figure 1 shows an example in which two isoforms, 1 and 2, share the same exon, C, which is connected to different upstream exons, A and B; therefore, we observe different ribosome profiles for these two isoforms, which is not considered by existing methods.

Another way to analyze ribosome profiles is to predict ribosome distributions according to transcript sequence. Liu and Song developed an effective model, RiboShape, by transforming the ribosome profiles into a low-dimensional space using a wavelet transformation and then building a sparse model to predict the transformed footprint [4]. Their predicted profiles are in a low-dimensional space from wavelet transformation, instead of at nucleotide-level resolution. This work inspired us to calculate ribosome distributions for transcripts by combination of allocating Ribo-seq reads to isoforms and predicting ribosome profile from isoform sequence.

Here, we present our model, called *DeepShape*, to analyze Ribo-seq data without the help of RNA-seq data. We introduced a deep learning ribosome distribution shape model to overcome heterogeneity of ribosome profiles by incorporating different upstream or downstream sequences as features to predict ribosome abundance. Multiple-mapped reads are allocated according to both pre-estimated ribosome abundances and distributions. Iteration mechanism was used for more accurate estimation of ribosome abundances and distributions for transcripts. Testing *DeepShape* on synthetic data shows that it can provide more accurate estimation on ribosome abundance and more precise ribosome distribution profiles than state-of-the-art methods. Using *DeepShape*, we analyzed a Ribo-seq dataset of the PC3 human prostate cancer cells [23] in transcript level. Isoforms of the four cell invasion/metastasis genes, which were reported to be regulated in translation by PP242 treatment in the original paper, show distinct translational efficiencies and regulation patterns. We proposed “Codon Residence Index” (CRI), which reflects the relative speed when a ribosome going through a codon compared with its synonymous codons. The CRI values between synonymous codons are significant. Several codons show at least 20% slower (higher-CRI) or faster (lower-CRI) in ribosome speed compared with the average of their synonymous codons in all the four experiments. After PP242 treatment, twelve codons represent consistently and statistically significant speed up or slowing down in both replicates. The observations validate the necessity of analyzing Ribo-seq data to produce transcript-level profiles.

## Implementation

### Synthetic data and real data used in this study

In this study, we generated a pair of synthetic Ribo-seq/RNA-seq sequence data to test the performance of our *DeepShape* method and other state-of-the-art methods (Additional file 1: Figure S1). To generate the synthetic Ribo-seq data, firstly we used published RNA-seq data from human HeLa cells (GSM546921) to calculate ground-truth transcript abundance. The transcript abundances are length-normalized, which is defined as

$$ {t}_i=\frac{c_{ti}/{l}_{ti}}{\sum_{k\in \mathbf{T}}\left({c}_{tk}/{l}_{tk}\right)}\times {10}^6, $$

where *c*_*ti*_ is the number of RNA-seq reads on transcript *i*, *l*_*ti*_ is the length of the *i*-th transcript, and **T** is the set of all transcripts. This index also has a name of “Transcripts Per Million (TPM)”, which is widely used in RNA-seq analysis. Human tRNA and rRNA reads were filtered out before clean reads were mapped to human HG38 transcriptome reference by STAR [24] and quantified by Salmon [25]. Secondly, we randomly generated a set of synthetic transcript efficiency (TE) values, which is the ratio of relative ribosome abundance over transcript abundance. The ribosome abundance values are normalized by coding region (CDS) length, and is defined as

$$ {r}_i=\frac{c_{ri}/{l}_{ri}}{\sum_{k\in \mathbf{T}}\left({c}_{rk}/{l}_{rk}\right)}\times {10}^6, $$

where *r*_*i*_ is the CDS-length normalized abundance, *c*_*ri*_ is the number of Ribo-seq reads on transcript *i* (ribosome count abundance), *l*_*ri*_ is the length of the coding region of the *i*-th transcript, and **T** is the set of all transcripts. And the TE values are *r*_*i*_/*t*_*i*_. TE values are generated following a log-normal distribution, thereby enabling calculation of Ribo-Seq reads counts. Thirdly, we calculated a synthetic ribosome distribution for every transcript using a ribosome flow model [26]. Finally, we generated the synthetic Ribo-Seq reads according to their reads counts, ribosome distribution and reference transcriptome, using the “footprint_generator” program from Ribomap. To generate synthetic RNA-seq data, we applied the rlsim program [27] on the TPM obtained above. The synthetic data generation pipeline is a simulation of the real translation and sequencing process.

To show the usefulness of our *DeepShape*, we also reanalyzed a published PC3 human prostate cancer cell dataset [23]. The dataset consists of two replicates of PP242-treated samples and two replicates of control samples. Each of these four samples was sequenced to generate Ribo-seq and RNA-seq data. We analyzed the isoform-level translation efficiency and potential translational regulatory events using this dataset. This dataset is called “PC3 dataset” in this study.

The accuracy of the shape model in *DeepShape* was compared to RiboShape, using the same dataset [4]. The original Ribo-seq data come from four published datasets of *S. cerevisiae* (yeast) treated with CHX [9, 28, 29]. Liu et al. (2016) calculated gene-level ribosome distribution using RiboShape. Genes with more than 10 continuous zero-read covered bases were discarded, resulting in 80 genes filtered out and a total of 2458 genes passing quality control. We randomly split the gene set into 3 subsets: 90% genes (2212) for training, 5% genes (124) for validation, and 5% genes (122) for testing. This dataset is called “yeast dataset” in this study. Besides, we also downloaded ribosome distribution data of three other model organisms (*E. coli* [30], mouse [31] and human [32]) from GWIPS (https://gwips.ucc.ie/) to test the performance of our shape model. As *E. coli* and yeast do not have alternative splicing, their datasets reflect real ribosome distribution on genes/transcripts. For alternatively spliced transcripts in mouse and human datasets, we simply joined the ribosome values in different exons to generate the ribosome distribution of transcripts.

### Designing of DeepShape-prime and DeepShape

Our method contains two separate programs. The first one is named *DeepShape-prime*, which can be used for fast estimating of ribosome abundances on transcripts without considering the heterogeneity of ribosome profiles from the same exons shared by different isoforms. *DeepShape-Prime* takes CDS-length-normalized ribosome abundance (see Section 2.1) as guidance of allocating multiple-map reads and updates the abundance iteratively.

**Fig. 2:**
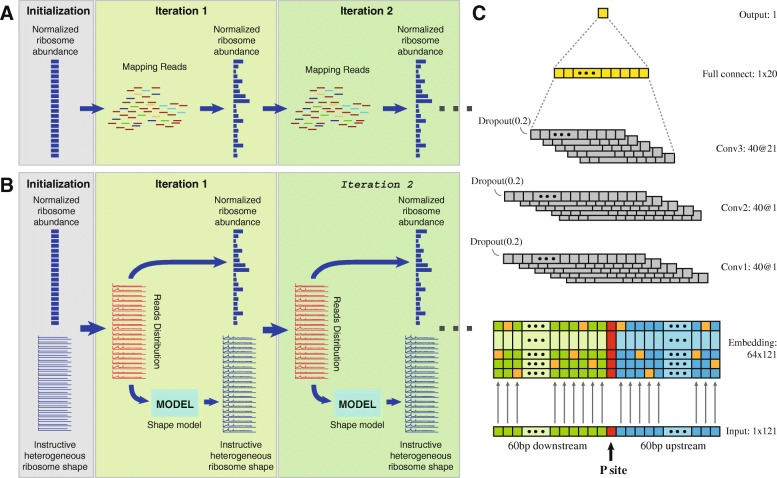
Designing of *DeepShape* and the shape model. **a** pipeline of *DeepShape-prime*. After a uniform initiation of ribosome abundance normalized by CDS length, in each iteration, reads are mapped proportional to normalized ribosome abundance, and then a new ribosome abundance is updated. **b** pipeline of *DeepShape*. A shape model is introduced, and in each iteration, reads are mapped proportional to ribosome abundance and an instructive ribosome shape together. Then the shape model is fitted to generate a new instructive ribosome shape which can introduce heterogeneity because of different contexts. **c** structure of the shape model. The model contains an embedding layer which converts the codons to ‘one-hot’ vectors, three convolutional layers and a full connection layer

As shown in Fig. 2a, initially, CDS-length-normalized ribosome abundance are set uniformly. Then, at each iteration, multiple-mapped reads are allocated proportionally to transcripts according to the current normalized ribosome abundance, and then, new ribosome abundance values are updated using the newest distributions of reads on transcripts.

The other program is called *DeepShape*, which differs from *DeepShape-prime* in that it aims to calculate more precise ribosome distributions along transcripts by allocating reads not only according to ribosome abundance, but also an instructive ribosome shape that were predicted from transcript sequences by a “shape model” that was trained from the Ribo-seq data being processed (Fig. 2b). At initiation, the normalized ribosome abundances are set uniformly, or given abundance values (say, from results of *DeepShape-prime*), and the instructive ribosome shapes are also set uniformly. In each iteration, *DeepShape* performs three tasks. At Step 1, it assigns reads according to current normalized ribosomal abundances and instructive ribosome shape values to obtain a temporary ribosome distribution. At Step 2, it trains the shape model using the temporary ribosome distributions and then applies the shape model to transcript sequences to update instructive ribosome shape values. At step 3, it updates a new set of ribosomal abundances according to the read abundance on the transcripts. This process iterates until the results are stabilized.

### Design and training of shape model

In *DeepShape*, the shape model is a context-dependent model which characterizes ribosome moving patterns or distributions. We use a convolution neural network (CNN) model to learn the association between the context nucleotides and shape of ribosome profiles (Fig. 2c). The model was built by a Python deep learning library Keras [33].

We used the encoded codon sequence, instead of nucleotide sequence, to represent the surrounding context of the ribosome. The input to the CNN model is the codon sequences of length 121 surrounding the position of ribosome (60aa upstream and 60aa downstream). The model contains a layer encoding 64 different codons into 64-dimensional ‘one-hot’ vectors, three convolutional layers, a full connecting layer, and an output layer. The output is the predicted instructive ribosome shape value at this site. The normalized values of ribosome distribution along transcripts were used here to train the deep learning model, which can be calculated as

$$ {x}_{ij}=\frac{c_{ij}}{\sum \limits_{j\in \left[1,{l}_i\right]}{c}_{ij}}\times {l}_i, $$

where *x*_*ij*_ denotes the normalized ribosome distribution of gene *i* at the position of its *j*-th codon, *c*_*ij*_ denotes the number of ribosome reads at position *j* on transcript *i*, and *l*_*i*_ denotes the length of the coding region of the transcript. Data with extremely high or low values (outside [*μ*-3*σ*, *μ* + 3*σ*] in log scale, *μ* and *σ* are the mean and standard deviation of all values in training data) or zeros are discarded from the training dataset. Besides, the original data follows a log-normal-like distribution. We did a data re-sampling to keep the data relatively balanced in each distance from center. The region between [*μ*-3*σ*, *μ* + 3*σ*] was divided into 10 bins, and in each bin, we re-sampled 50,000 data points.

We train the shape model by minimizing the following loss function:

$$ L=\frac{\sum_{i=\left[1,N\right]}{\left[\log \left(\max \left({10}^{-8},{y}_{predict}\right)\right)-\log \left(\max \left({10}^{-8},{y}_{ground- truth}\right)\right)\right]}^2}{N}, $$

where *N* denotes the size of the training data. The training of the CNN model stops if the loss of the validation set in current epoch is higher than that of the last epoch.

### Comparison of the shape model and *DeepShape*

We applied the shape model of *DeepShape* to datasets from four different organisms downloaded from GWIPS: the yeast dataset used in RiboShape [4], an *E. coli* dataset [30], a mouse dataset [31] and a human dataset [32]. We compared our performance with that of RiboShape.

We compared both *DeepShape-prime* and *DeepShape* with Ribomap for accuracy of the predicted ribosome distributions at the transcripts. Default parameters of Ribomap were used in this study. We also included a naive method which distributes the multiple-mapped reads uniformly to all target transcripts. As the mapping process was carried out by STAR [24], we call the naïve method as “STAR-uniform” in this paper. In addition, we tested an RNA-seq quantification tool, Salmon, using the synthetic Ribo-seq data directly, in order to assess the performance of the representative RNA-seq profiling method.

### Calculation of codon residence index

To study potential translation mechanisms, we investigated the translation speed of codons. We defined Codon Residence Index (CRI), which shows the relative speed when a ribosome going through a codon compared with its synonymous codons. This index is defined in the following:

$$ {\mathrm{CRI}}_{ij}=\frac{\frac{1}{n_{ij}}{\sum}_{k=1}^{n_{ij}}{x}_{ik}^j}{\frac{1}{\#{\mathbf{S}}_j}{\sum}_{j^{\prime}\in {\mathbf{S}}_j}\frac{1}{n_{i{j}^{\prime }}}{\sum}_{k=1}^{n_{i{j}^{\prime }}}{x}_{ik}^{j^{\prime }}}-1, $$

where CRI_*ij*_ denotes the CRI value of codon *j* on a specific transcript *i*, ${x}_{ik}^j$ denotes the normalized ribosome distribution value (see Section 2.3) at the position of the *k*-th codon *j* on the *i*-th transcript, *n*_*ij*_ denotes the number of codon *j* on transcript *i*, and **S**_*j*_ denotes the set of codon *j*’s synonymous codons on that transcript (including *j*). If a codon has a CRI value above zero, it means that a ribosome tends to pay more time on this codon compared with its synonymous codons. On the contrary, a CRI value below zero means that a ribosome tends to move faster on the codon compared with its synonymous codons.

## Results

### Performance of the shape model on benchmark datasets

To assess the accuracy of ribosome shape prediction, we applied the aforementioned computational models to the yeast dataset (see Methods). Table 1 shows the average Pearson correlation coefficients (PCC) between predicted ribosome profiles and ground-truth profiles for genes with different coding length. Our CNN model in *DeepShape* can produce an average PCC of 0.60 in the testing dataset (Additional file 1: Figure S2). Genes from 251 aa to 500 aa in length tend to have the most accurate predictions. Liu et.al. showed that prediction on lower wavelet transformation space can give higher correlations using their method. Even so, our results show a better performance (0.57~0.63 PCC) without any smoothing procedure than that of RiboShape on smoothed V7 space (0.46~0.52 PCC), and even on very smoothed V4 space (0.49~0.62 PCC) (Table 1). These results prove the advantage of our CNN model.

**Table 1:** Performance of shape model on genes with different lengths

Length (codons)	RiboShape V3^a^	RiboShape V4^a^	RiboShape V7^a^	Shape model *(DeepShape)*
<=250	0.61	0.53	0.45	0.61
251–500	0.67	0.62	0.52	0.63
501–750	0.61	0.58	0.50	0.59
751–1000	0.52	0.49	0.46	0.57
> 1001	0.54	0.51	0.46	0.58

^a^Reported in RiboShape study [4]. The V3 and V4 spaces are highly smoothed subspaces of the original ribosome distribution profile after wavelet transformation. V3 is smoother than V4. The V7 space is also smoothed, but most similar to the original space. RiboShape performs better in a lower subspace

We also tested our shape model and RiboShape on ribosome distribution datasets of *E. coli*, mouse and human (Methods and Additional file 1: Supplementary Methods). In *E. coli* and human datasets, our shape model outperforms RiboShape (Additional file 1: Table S1). In the mouse dataset, the two methods gave similar results. However, the predicted ribosome shape that our shape model provides is at the codon level resolution, while RiboShape can only give smoothed prediction in subspace. Moreover, our shape model has great advantage in computational time. For example, on the *E. coli* dataset, our shape model took less than 2 hours running a 10-fold cross validation, while RiboShape took more than 3 days.

### Performance of ribosomal abundance estimation using synthetic datasets

We simulated a pair of synthetic Ribo-seq/RNA-seq dataset, both containing 20,000,000 reads with length 30 bps (Additional file 1: Supplementary Methods). The read length and mRNA abundance were set following a real dataset from human HeLa cells [34] (see Section 2.1), which was also used in the study of Ribomap [15]. We applied *DeepShape*, *DeepShape-prime* and Ribomap to this dataset to show their performances in predicting ribosome abundance. *DeepShape* and *DeepShape-prime* used Ribo-seq data only, while Ribomap used both Ribo-seq data and RNA-seq data. Both ribosome count abundance and CDS-length-normalized abundance (see Section 2.1) were evaluated and compared.

**Fig. 3:**
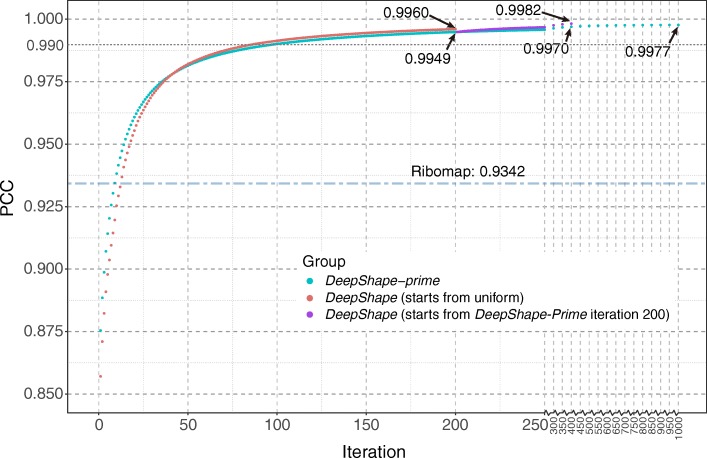
PCC curves for prediction of ribosome abundance at each iteration of *DeepShape* and *DeepShape-prime*. For comparison, the PCC value of Ribomap is shown

Figure 3 plots the PCC curves of ribosome count abundance (measured in absolute counts of ribosome-protected fragments on CDS regions) estimated by *DeepShape-prime*, *DeepShape* and Ribomap, compared with the “ground-truth” of preset ribosome abundance in the synthetic dataset. *DeepShape-prime* was run for 1 to 1000 iterations (blue dots) and *DeepShape* was run for 1 to 200 iterations. For *DeepShape*, two kinds of initialing ribosome abundance (see Section 2.2 in Methods) were tested: uniform (red dots), or direct results from the 200-th iteration of *DeepShape-prime* (purple dots).

We observe that the PCC value of *DeepShape-prime* improves quickly at the first 30 iterations, becoming stabilized, and then finally converging to values higher than 0.99 after 100 iterations. The convergent speed of *DeepShape* is a bit shower than that of *DeepShape-Prime* at the beginning iterations because it has an additional module for shape prediction, which at the end leads to higher accuracy than *DeepShape-prime* (0.9960 vs. 0.9949, in the 200-th iteration). Both *DeepShape* and *DeepShape-prime* outperform Ribomap (PCC 0.9342, blue dots-dashed line) after about 10 iterations. Starting from ribosome abundance exported from *DeepShape-prime* can help *DeepShape* get better performance compared with uniform initialization (0.9982 vs 0.9960, both in the 200-th iteration). To test the accuracy of existing isoform-level RNA-seq quantification algorithms on Ribo-seq data, we applied Salmon, a widely used program, to the same dataset, and obtained a PCC value of only 0.8923, much worse than existing Ribo-seq programs. This shows that designing computational tools specifically for Ribo-seq data analysis is necessary. The performances of estimating length-normalized ribosome abundances by *DeepShape-prime*, *DeepShape* and Ribomap show similar results (Additional file 1: Figure S3). In practice, as *DeepShape-prime* runs much faster because it does not need to calculate the shape, and since the distinction between *DeepShape* and *DeepShape-prime* is quite small, user can choose *DeepShape-prime* for efficient estimation for ribosome abundance information.

Additional tests were conducted to evaluate the robustness of *DeepShape-prime*. We tested *DeepShape-prime* with 200 iterations against Ribomap on three randomly generated synthetic datasets using several measures. Table 2 shows that *DeepShape-prime* outperforms Ribomap in all three datasets. The performance of *DeepShape-prime* stabilizes at values higher than 0.99 for ribosome abundance PCC, yet the performance of Ribomap floats from around 0.87 to 0.93. The ribosome loading MSE values of *DeepShape-prime* are about one magnitude smaller than those of Ribomap. Moreover, from the aspect of CDS-length-normalized ribosome abundance, we observe that the performance of Ribomap relies a lot on the extent of similarity between mRNA abundance and ribosome abundance (last three lines in Table 2). The results show that *DeepShape* is more robust than Ribomap.

**Table 2:** Ribosome abundance estimation performance in three replicate synthetic datasets

Evaluation	Method	Dataset 1	Dataset 2	Dataset 3
Ribosome count PCC	*DeepShape-prime*	0.9931	0.9947	0.9932
	Ribomap	0.8952	0.9253	0.8680
Ribosome count MSE	*DeepShape-prime*	87,546	70,815	157,187
	Ribomap	1,216,917	854,546	2,213,367
CDS-Length-normalized ribosome abundance PCC	*DeepShape-prime*	0.9990	0.9991	0.9981
	Ribomap	0.9158	0.9381	0.9616
Ground-truth mRNA vs ribosome abundance PCC (both normalized by length)		0. 6775	0.7162	0.8252

*PCC:* Pearson correlation coefficient between prediction and ground-truth.

*MSE:* Mean square error between prediction and ground-truth.

### Performance of ribosomal shape prediction

To evaluate the performance of ribosome shape prediction, we compared the mean shape Pearson correlations of the following four models: *DeepShape-prime*, *DeepShape*, Ribomap, and a simple model, called STAR-uniform, which allocates multiple-mapped reads randomly to their mapped genes. The methods were applied and evaluated in our synthetic dataset (see Section 2.1).

**Fig. 4:**
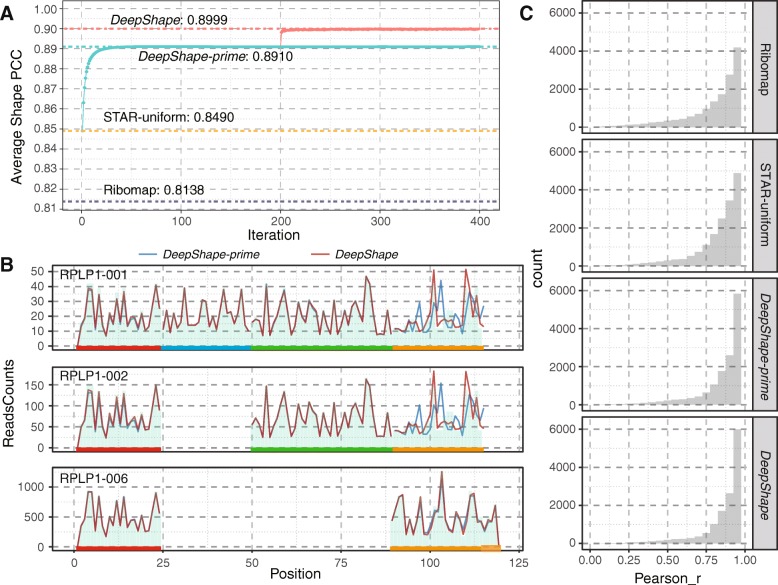
Improvement after introducing shape model to *DeepShape*. **a** Changing of ribosome profile shape PCC along the iteration of *DeepShape-prime* and *DeepShape*. The best performances are provided as dash dot lines (red: *DeepShape*, green: *DeepShape-prime*, yellow: STAR-uniform, purple: Ribomap). **b** An example of ribosome profiling improvement by *DeepShape* (red line) compared to *DeepShape-prime* (blue line). Ground-truth is shown as light green bars. The RPLP1 gene has three isoforms, including four exons (red, blue, green and yellow lines below the profile). *DeepShape* tends to provide the same profile in the same exons. **c** Distributions of isoform PCCs between calculated ribosome distributions and ground-truth

Figure 4a shows the PCC curves of the ribosome shape predictions for both *DeepShape-prime* and *DeepShape* from iterations 1 to 400, as well as the PCC values by Ribomap and STAR-uniform. *DeepShape* has the best average shape PCC of 0.900, followed by *DeepShape-prime* with 0.891. The result of the STAR-uniform is 0.849, but better than that of Ribomap (0.814). This is because of the misleading information by using RNA-seq abundance, which may be very different from ribosome abundance because of variation of translation efficiency. It should be noted that *DeepShape* first obtained the ribosome abundance results of the 200th iteration of *DeepShape-prime* and then estimated the ribosome profiles, which improved the PCC value from 0.891 to 0.899 after about 10 iterations.

We observed a noticeable improvement of ribosome shape predictions for many genes. For example, from STAR-uniform to *DeepShape*, more than 3405 genes have a better profile with improved PCC value > 0.1. The “small” improvement from *DeepShape-prime* (0.891) to *DeepShape* (0.900) means that 421 genes have a better profile with improved PCC value > 0.1.

Figure 4b shows an example of reconstructed ribosome profiles by *DeepShape*, *DeepShape-prime*, Ribomap and STAR-uniform. The three transcripts come from gene RPLP1 (ribosomal protein, large, P1. Gene ID: ENSG00000137818). This gene contains four exons, and because the length of Exon 3 is not a multiple of three, a frame shift on Exon 4 occurs in transcript RPLP1–006 compared with transcripts RPLP1–001 and RPLP1–002; hence, the ribosome distribution at this exon varies by transcripts. After introducing the shape model, we observe that the ribosome distribution of RPLP1–001 and RPLP1–002 improves significantly compared to the “ground-truth” when generating the synthetic data (Fig. 4b, light green bars) from *DeepShape-prime* (Fig. 4b, blue line) to *DeepShape* (Fig. 4b, red line) by the increase of PCC value of RPLP1–001 from 0.828 to 0.966 and the increase of PCC value of RPLP1–002 from 0.795 to 0.961. The improvement solely results from better ribosome distribution prediction. *DeepShape-prime* allocates multiple-mapped reads proportionally according to ribosome abundance; therefore, in its results, the ribosome distribution shapes on exon 4 are similar among the three transcripts, as the proportions are dominated by the one with highest abundance (the third transcript here). In contrast, the *DeepShape* model catches the frame shift in computing the ribosome profile.

Figure 4c shows the distributions of Pearson correlations between predicted gene profiles and the ground-truth. Results show that *DeepShape* has significantly higher PCC values compared to those of both Ribomap and STAR-uniform. Ribosome profiles of more than 68.9% (13,823 out of 20,073) of isoforms can be reconstructed with Pearson correlation > 0.9 by *DeepShape-prime*.

### Translation process revealed by DeepShape

#### Discovery of ribosomal stalling using synthetic datasets

We demonstrate that *DeepShape* can be used to discover ribosomal stalling events, defined as the positions at which ribosome profiles are higher than μ + 2σ, where μ and σ are the mean and standard deviation of the normalized ribosome distribution. We first annotated stalling events in the ground-truth ribosome profiles of the synthetic dataset. We then applied *DeepShape*, *DeepShape-prime*, Ribomap and STAR-uniform to the synthetic data, respectively, obtaining the ribosome profiles, followed by annotation of stalling events on the reconstructed profiles. Additional file 1: Figure S4 shows the results. Out of the 134,137 stalling events annotated as the ground-truth, *DeepShape* could identify 87.7% (117,647), higher than Ribomap (65.5%), STAR-uniform (74.4%) and *DeepShape-prime* (87.1%), showing the high sensitivity of DeepShape. Moreover, *DeepShape* has the highest precision (84.1%), compared to Ribomap (64.3%), STAR-uniform (68.3%) and *DeepShape-prime* (83.6%). All these results validate that *DeepShape* can accurately identify more translation events.

#### Calculating translation efficiency with Ribo-seq

We re-analyzed a Ribo-seq dataset of human prostate cancer cells (PC3 dataset) [23]. In cancer development, mammalian target of rapamycin (mTOR) kinase is a very important factor [35, 36], and an mTOR ATP site inhibitor, PP242, can inhibit the downstream pathway of mTOR. The effectiveness of PP242 (an mTOR ATP site inhibitor) on downregulating the translation of mTOR sensitive genes in PC3 cells was proved in gene level [23]. Here using *DeepShape*, we analyzed the translational response to PP242 treatment in transcript level.

**Fig. 5:**
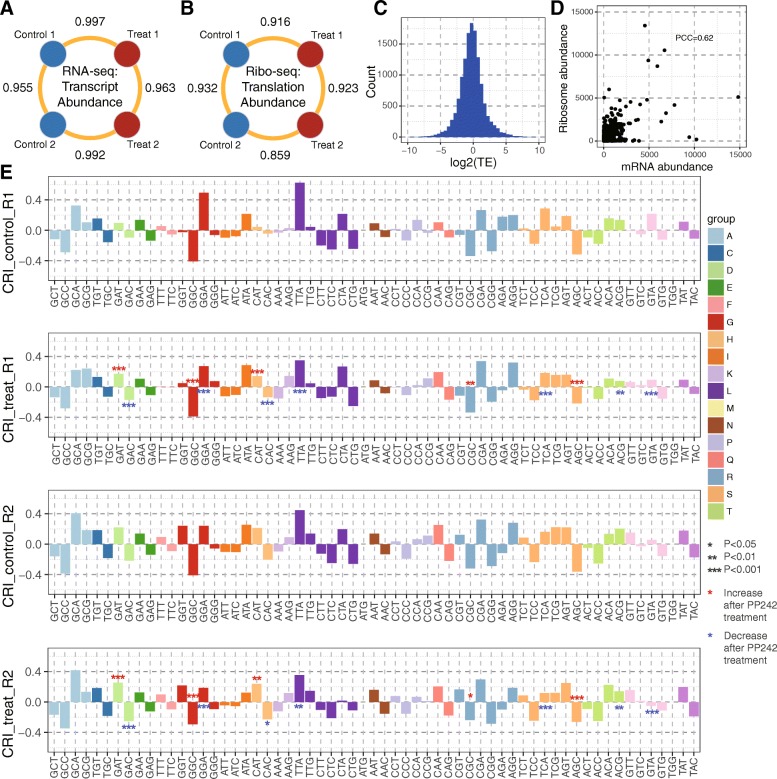
Application of *DeepShape* to reveal translational regulation patterns. **a**/**b** mRNA/ribosome abundance PCCs between and inside conditions. **c** distribution of TE in log 2 scale. **d** relation between length-normalized mRNA and ribosome abundance. The overall PCC between mRNA and ribosome is 0.62. **e** Codon Residence Index (CRI) of different codons in control of Replicate 1 (row 1), treat of Replicate 1 (row 2), control of Replicate 2 (row 3) and treat of Replicate 2 (row 4). The values show average CRIs of all transcripts that with length-normalized mRNA and ribosome abundance higher than 10. Significances between control and treat were tested by paired samples Wilcoxon two-sided test

We first computed the mRNA and ribosome abundance correlations (evaluated in PCC) for samples at the same condition and between treatments (Fig. 5b and c). High consistency was observed between two replicates under the same condition (control or PP242 treatment). Interestingly, for mRNA abundance, the correlation between treatments are even higher than that in the same condition (Fig. 5a), indicating that the treatment did not significantly alter expression of transcripts. On the other hand, for ribosome abundance, the correlation between the two conditions is higher (Fig. 5b). After PP242 treatment, these observations show that the change of translating ribosome abundance is higher than the change of gene expression.

We analyzed the translation efficiencies (TE) in each experiment. In previous works, TE was mainly calculated by ribosome reads/RNA-seq reads [9, 23], or with some self-defined normalization method [15]. Although these approaches can reveal the relative efficiency when comparing different isoforms, the results are easily affected by sequencing depth. Here we use the length-normalized ratio (of ribosome abundance/mRNA abundance) to define TE (see Section 2.1), which follows a log-normal distribution (Fig. 5c). About a quarter of transcripts (5311 out of 19,522) have a translation efficiency value higher than 4 or lower than 0.25. It is obvious that mRNA abundance is very different from ribosome abundance (Fig. 5d). This also confirms that allocating multiple-mapped reads by RNA-seq transcript abundance is not suitable.

In the original paper of the PC3 dataset, four cell invasion/metastasis genes were reported to be significantly regulated in translation by PP242 treatment: *VIM*, *YB1*, *MTA1*, and *CD44*. This gene level observation is confirmed in our study on transcript level (Table 3). Meanwhile, we found that isoforms of the same gene may have very different TE or TE regulation patterns (Table 3). For example, four isoforms were found in *VIM*: VIM-001, VIM-002, VIM-003 and VIM-201 (Additional file 1: Figure S5). Among them, VIM-001 and VIM-201 have the same protein coding region, and we therefore combined them into one. VIM-002 and VIM-003 are alternatively spliced transcripts and annotated as nonsense-mediated decay (NMD), which is regarded as erroneous splicing and will be decayed under normal circumstances. In the two replicates of experiments, VIM-001 (VIM-201) shows similar TEs and TE regulation patterns after PP242 treatment, and the two NMD transcripts show very different TEs but similar TE regulation patterns (Table 3). Both NMD transcripts of VIM genes show lower TEs in replicate 2, and the effect of down-regulation by PP242 is more obvious than that of replicate 1 (Additional file 1: Table S2). The *MTA1* gene also have similar phenomenon. The isoform switching also occurs in other cell types (Additional file 1: Table S3). Because of the lack of isoform-level translation studies in the past, in-depth study on the difference of TE among isoforms from the same gene is absent from the literature. In view of this, the analysis here may provide a new paradigm for the study of translation.

**Table 3:** mRNA and ribosome abundance in the four cell invasion/metastasis genes

Gene	Transcript	mRNA (TPM)				Ribosome (CDS-length-normalized)			
		control1	control2	treat1	treat2	control1	control2	treat1	treat2
*VIM*	ENST00000544301.6 (VIM-001)	1268.151	1079.626	1864.131	1606.06	1530.699	1432.324	655.713	491.6958
	ENST00000487938.5 (VIM-002)	1.19409	5.19589	4.60853	6.37172	3.314905	1.386528	2.49548	1.02E-20
	ENST00000469543.5 (VIM-003)	8.6292	9.6432	5.48475	4.47247	189.396	13.71204	42.24884	0.044353
*YBX1*	ENST00000321358.11 (YBX1–201)	932.746	837.964	1007.04	921.105	641.5486	579.1333	228.5892	145.99
*MTA1*	ENST00000331320.11 (MTA1–001)	7.72171	0	9.44299	7.80098	3.139666	0.0147	0.012351	1.39E-13
	ENST00000405646.5 (MTA1–003)	49.2963	38.3313	43.2853	35.8685	16.53584	18.04329	18.76716	7.94493
	ENST00000438610.5 (MTA1–004)	10.3167	7.68285	14.4819	14.1276	6.170347	5.191197	8.055225	6.377454
*CD44*	ENST00000263398.10 (CD44–003)	68.0735	74.2861	77.4636	74.5578	241.7872	311.4829	187.2566	177.6087
	ENST00000434472.6 (CD44–008)	3.81167	5.32514	8.42678	10.1775	9.713445	13.79658	7.088849	13.68229

#### Translation process analyzed by combining RNC-seq with Ribo-seq

Codon usage is one of the main factors related to the speed of ribosome movement along transcripts [37]. The speed of translating a codon is supposed to be related to the current cellular concentration of its corresponding tRNA [38, 39]. As outer environmental stress may affect tRNA concentration [40], it may also affect codon-specific ribosome movement speed. Here we designed an index, named “Codon Residence Index” (CRI, see Section 2.5), which stands for the relative time usage when a ribosome passing by a codon comparing with its synonymous codons. Using CRIs calculated from the ribosome distribution reported by *DeepShape*, we analyzed the association of PP242 treatment and ribosomes’ movement in codon-resolution.

To avoid inaccurate ribosome distribution estimation per codon on isoforms with low abundance, we only considered isoforms with both length-normalized ribosome and mRNA abundance higher than 10. Figure 5e shows the 64 codons’ average CRI values among all filtered transcripts. The four PC3 experiments show very similar CRI patterns (Fig. 5e). It’s common that a codon shows obvious speed up (lower CRI) or pause (higher CRI) compared with its synonymous codons. For example, the ribosome’s resident times on GCC, GGC, CGC and AGC are at least 20% shorter than on their synonymous codons (CRI ≤  − 0.2), in all the four experiments. On the contrary, the ribosomes averagely stay at least 20% longer on GCA, TTA and CGA than on their synonymous codons (CRI ≥ 0.2).

CRI patterns of the same replicate are more similar compared with that of the same condition (treat/control), which imply that each cell has their relatively characterized CRI pattern. However, after PP242 treatment, the trend of CRI shifts of the two replicates shows some interesting phenomenon: five codons show significant ribosome speed up in both replicates, and on the contrary, seven codons show significant ribosome slowing down. For all the four codons with CRI lower than −0.2 (GCC, GGC, CGC and AGC), the ribosome resident time on three of them (GGC, CGC and AGC) were significantly increased (Fig. 5, red stars) in both replicates. The other codon, GCC, also has increased average CRI values in both replicates after PP242 treatment. This implies potential regulatory mechanism of outer environment stress on codon-specific translation.

## Discussion

In this paper, we developed a computational method, *DeepShape*, which not only accurately estimates ribosome abundance at isoform level, but also provides precise ribosome distribution profiles along transcripts. *DeepShape* uses Ribo-seq reads only, making full use of multiple-mapped reads.

Many excellent RNA-seq quantification tools for isoform-level abundance estimation are available, such as cufflinks [41], RSEM [42], Salmon [25] and eXpress [43]. However, Ribo-seq data have characteristics that are very different from those of RNA-seq data. For example, the location of Ribo-seq reads is actually in the upstream of real ribosome binding site, and most Ribo-seq reads are targeted to the CDS region. The main source of bias of Ribo-seq reads comes from the movement of ribosomes, not RNA-seq sequence bias. For all these reasons, existing RNA-seq tools cannot be directly applied to Ribo-seq data analysis.

The movement of ribosome is highly non-uniform, but at exon-level, the overall non-uniformity of coverage can be smoothed. Compared with allocating multiple mapped reads according to mRNA abundance, *DeepShape* is a more accurate approach to making use of all mapped reads. Results on synthetic data proved its superiority over the state-of-the-art methods, and the analytic results for a published human prostate cancer cell dataset show many novel observations of cancer-related, isoform-level translational regulation events, which may point to new biological discoveries.

*DeepShape* was developed for model organisms that have complete and accurate transcript annotations. For non-model organisms with incomplete or erroneous transcript annotations, the accuracy of our model will be affected. We hope to investigate this topic in the future.

Some parameters in *DeepShape* can be adjusted by users. For example, the input length of the shape model used in this study is 121 codons. However, users can manually set this number to other lengths. According to the sequence depth of Ribo-seq dataset, users can also adjust the bin number and data number in the re-sampling step when training the shape model. The number of iterations in running *DeepShape-prime* and *DeepShape* can also be defined by users. For faster computation, user can set a smaller number.

In this study, we also proposed a codon-resolution index, Codon Residence Index (CRI) to study the relative ribosome moving speed on each codon compared with its synonymous codons. Using the ribosome distribution given by *DeepShape*, we found some interesting CRI pattern in human PC3 cells, including potential regulation pattern that related with PP242 treatment. However, the mechanisms of CRI patterns need to be further investigated. For example, we’ve also calculated the CRI patterns of a human A549 cell and a human HBE cell [20, 22]. Results show that the two cell-lines has very similar CRI pattern, yet distinct from the patterns of the PC3 cells in this study (Additional file 1: Figure S6). This implies that the CRI pattern may not be cell-line-specific, but rather related to different experimental protocols. The suggestion also reminds us to think about the necessity of considering and eliminating the experiment-specific bias when reading the underlying translation regulation mechanisms from ribosome distribution data.

## Conclusion

We propose *DeepShape* to analyze Ribo-seq data, and estimate ribosome abundance and distribution at isoform level. *DeepShape* makes full use of multiple-mapped reads. Compared with another state-of-art method, Ribomap, *DeepShape* needs only Ribo-seq data, without any RNA-seq data. *DeepShape* can help researchers to understand translation mechanisms.

## Supplementary information

**Additional file 1: **Supplementary Methods. **Figure S1.** Pipeline for generating synthetic data. **Figure S2.** Distribution of Pearson correlation coefficients (PCC) between predicted ribosome distributions by the shape model in *DeepShape* and the ground-truth in training, validation and testing datasets. **Figure S3.** PCC curves for prediction of length-normalized ribosome abundance at each iteration of *DeepShape* and *DeepShape-prime*. **Figure S4.** Application of *DeepShape* to annotating ribosome stalling events. **Figure S5**. Four isoforms of the *VIM* gene. **Figure S6.** Codon Residence Index (CRI) of different codons in A549 and HBE cell. The data are from the work of Lian et al. (Lian, et al., 2016). **Figure S7.** Diagrammatic explanation of finding P-sites. **Table S1.** Performance of shape model on genes with different lengths in *E. coli*. **Table S2.** TE and TE fold changes after PP242 treatment in the four cell invasion/metastasis genes. **Table S3.** mRNA abundance, ribosome abundance and TEs in the four cell invasion/metastasis genes of A549 and H1299 cells.

## Data Availability

Source codes: https://github.com/cuihf06/DeepShape. (This is an open source software for all academic or non-academic users. Operating system: Linux; Programming language: Python.) Ribosome shape data of yeast, *E. coli*, mouse and human: https://gwips.ucc.ie/ Human HeLa RNA-seq data for synthetic data generation (Gene Expression Omnibus): GSM546921. Human real PC3 data (Sequence Read Archive): SRR403882, SRR403883, SRR403886-SRR403889, SRR403892, SRR403893.

## Abbreviations

CDS: Coding sequence
CNN: Convolution neural network
CRI: Codon residence index
mTOR: Mammalian target of rapamycin
PCC: Pearson correlation coefficients
Ribo-seq: Ribosome sequencing
RPFs: Ribosome-protected fragments
TE: Transcript efficiency
TPM: Transcripts per million
