# Supplementary material for: DeepShape: estimating isoform-level ribosome abundance and distribution with Ribo-seq data
Source: BMC Bioinformatics. 2019 Dec 20;20(Suppl 24):678. doi: 10.1186/s12859-019-3244-0 (PMC6923924; doi:10.1186/s12859-019-3244-0)
Supplement: Supplementary file 1 — Additional file 1: Supplementary Methods. Figure S1. Pipeline for generating synthetic data. Figure S2. Distribution of Pearson correlation coefficients (PCC) between predicted ribosome distributions by the shape model in DeepShape and the ground-truth in training, validation and testing datasets. Figure S3. PCC curves for prediction of length-normalized ribosome abundance at each iteration of DeepShape and DeepShape-prime. Figure S4. Application of DeepShape to annotating ribosome stalling events. Figure S5. Four isoforms of the VIM gene. Figure S6. Codon Residence Index (CRI) of different codons in A549 and HBE cell. The data are from the work of Lian et al. (Lian, et al., 2016). Figure S7. Diagrammatic explanation of finding P-sites. Table S1. Performance of shape model on genes with different lengths in E. coli. Table S2. TE and TE fold changes after PP242 treatment in the four cell invasion/metastasis genes. Table S3. mRNA abundance, ribosome abundance and TEs in the four cell invasion/metastasis genes of A549 and H1299 cells. [file 12859_2019_3244_MOESM1_ESM.docx]

**Supplementary Methods**

**1. Preprocessing of reference databases**

We used two kinds of references in this study. One helps filter human tRNA and rRNA away from RNA-Seq and Ribo-seq sequences (hereinafter termed “non-coding reference”), and the other is a human transcriptome reference in Release 26.

To establish the “non-coding reference”, we selected human rRNAs from a human ncRNA database (<http://asia.ensembl.org/info/data/ftp>) and selected human tRNA sequences from the reference of eukaryotic tRNAs (<http://gtrnadb.ucsc.edu/download/tRNAs/>).

The human transcript sequences were downloaded from the GENCODE website (<https://www.gencodegenes.org/releases/26.html>). Finally, we performed quality control on the transcript reference sequences according to the “protein-coding transcript sequences” and the “comprehensive gene annotation” file downloaded from GENCODE in order to filter transcripts potentially wrongly annotated or too short. We excluded the following transcripts:

1. Transcripts inconsistent with the translated peptides (translated peptide sequence is different from the reference of protein-coding sequences);
2. Transcripts with stop codon in the middle of CDS region (in the same reading frame of the start codon in the transcript);
3. Transcripts with duplicated sequences (two or more sequences with the same transcript ID);
4. Peptide sequence length less than 3 aa;
5. Transcripts without a start codon (a single-nucleotide substitution on AUG was allowed, neglecting other alternative start codons);
6. Transcripts the CDS of which does not stop with a stop codon.

**2. Ribosome abundance and distribution estimation**

First, adapters were removed from original Ribo-seq reads. Trimmed reads were in multiple lengths which presents RPFs with different length. We then mapped the trimmed Ribo-seq reads to the human “non-coding reference”, followed by alignment of unmapped reads to the human transcriptome reference. We used the same mapping strategy as that used in Ribomap to make the results comparable (Wang, et al., 2016). Reads with 25~36bp length were kept and STAR was used for alignment.

Second, the “P-site” position was inferred according to read length. Ribosome is characterized by three mRNA binding sites: A-site for “arrival”, P-site for “polypeptide” and E-site for “exit” (Liu and Song, 2016). The P-site helps to recognize the codon and hold corresponding tRNA, thus extending a new peptide. The length of a Ribo-seq read is often around 30bp (25bp~36bp for most reads), and the P-site is often at the middle of the read. We followed the way by Ribomap (Wang, et al., 2016) to decide the positon of P-sites, to make sure that the comparison between DeepShape and Ribomap is fair. Briefly, for each read that representing a ribosome on transcript, we inferred its P-site position by finding the position of its “P-site mark” (Supplementary Fig. S7), which is the 13th nucleotide for read length between 25bp and 30bp, 14th nucleotide for read length between 31bp and 33bp, and 15th nucleotide for read length between 34bp and 36bp. For most situations, the “P-site mark” would hit the first nucleotide of a codon, yet there still exists a portion of “P-site marks” hit the second and third nucleotide (Calviello and Ohler, 2017). In this study, if the “P-site mark” of an RPF read hit any position of a codon, this codon was then considered as the P-site of the ribosome.

Finally, *DeepShape* or *DeepShape-prime* takes as input the P-site adjusted alignment results and produces a list of CDS-length-normalized ribosome abundance values and ribosome read distributions on transcripts. *DeepShape* can also produce a shape model with trained parameters.

**3. Analysis of RNA-seq and RNC-seq data**

Similar to the first step of Ribo-seq read processing, the RNA-seq and RNC-seq sequences were first filtered by “non-coding reference” and then mapped to the human transcriptome database. Both steps are carried out by STAR (Dobin, et al., 2013). The bam results were quantified by Salmon (Patro, et al., 2017) with bias correction.

**4. Other statistical analysis**

We evaluated ribosome abundance estimation performance of the aforementioned computational models by Pearson correlation coefficients (PCC) between the estimated abundances and the ground-truth.

To evaluate ribosome distribution along transcripts, we calculated the PCC value between the estimated ribosome distributions and the ground-truth for each method, followed by comparing the methods by plotting the PCC distribution of the whole transcriptome and by averaging PCC scores.

For the RNC and PC3 datasets, we only considered isoforms with both relative length-normalized ribosome and mRNA abundance higher than 0.1, thus avoiding inaccurate abundance estimation on isoforms with low abundance.


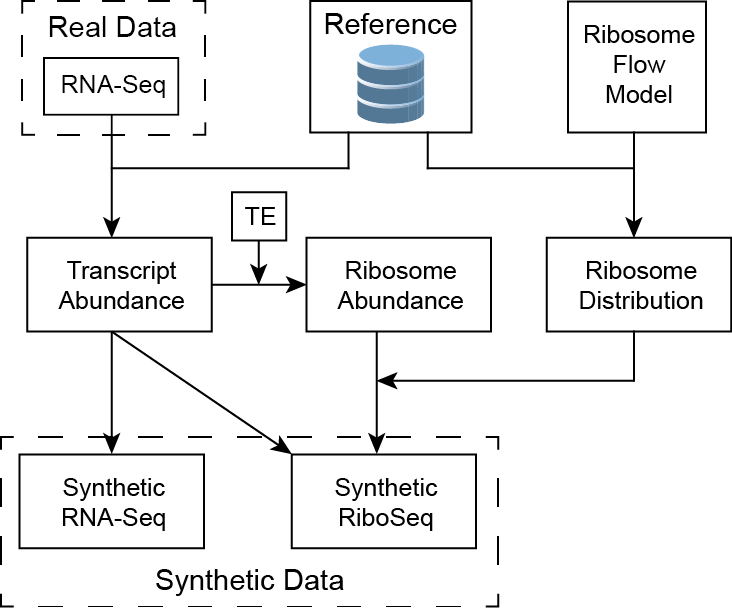


**Figure S1. Pipeline for generating synthetic data.** Transcript abundance was first obtained from a real RNA-Seq dataset, and translational efficiency (TE) was generated using a log-normal distribution. Then ribosome abundance was calculated from TPM and TE, and the synthetic ribosome distribution along transcripts was generated from a ribosome flow model. Finally, synthetic RNA-Seq data and Ribo-seq data were generated.


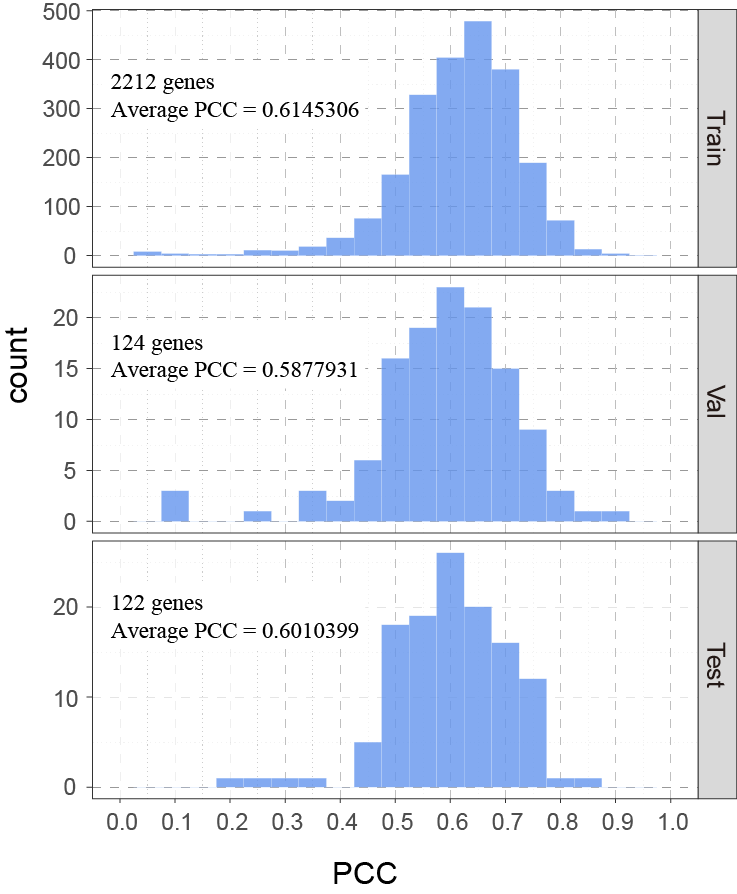


**Figure S2. Distribution of Pearson correlation coefficients (PCC) between predicted ribosome distributions by the shape model in *DeepShape* and the ground-truth in training, validation and testing datasets.** The shape model gives an average of 0.60 PCC value on the testing data.


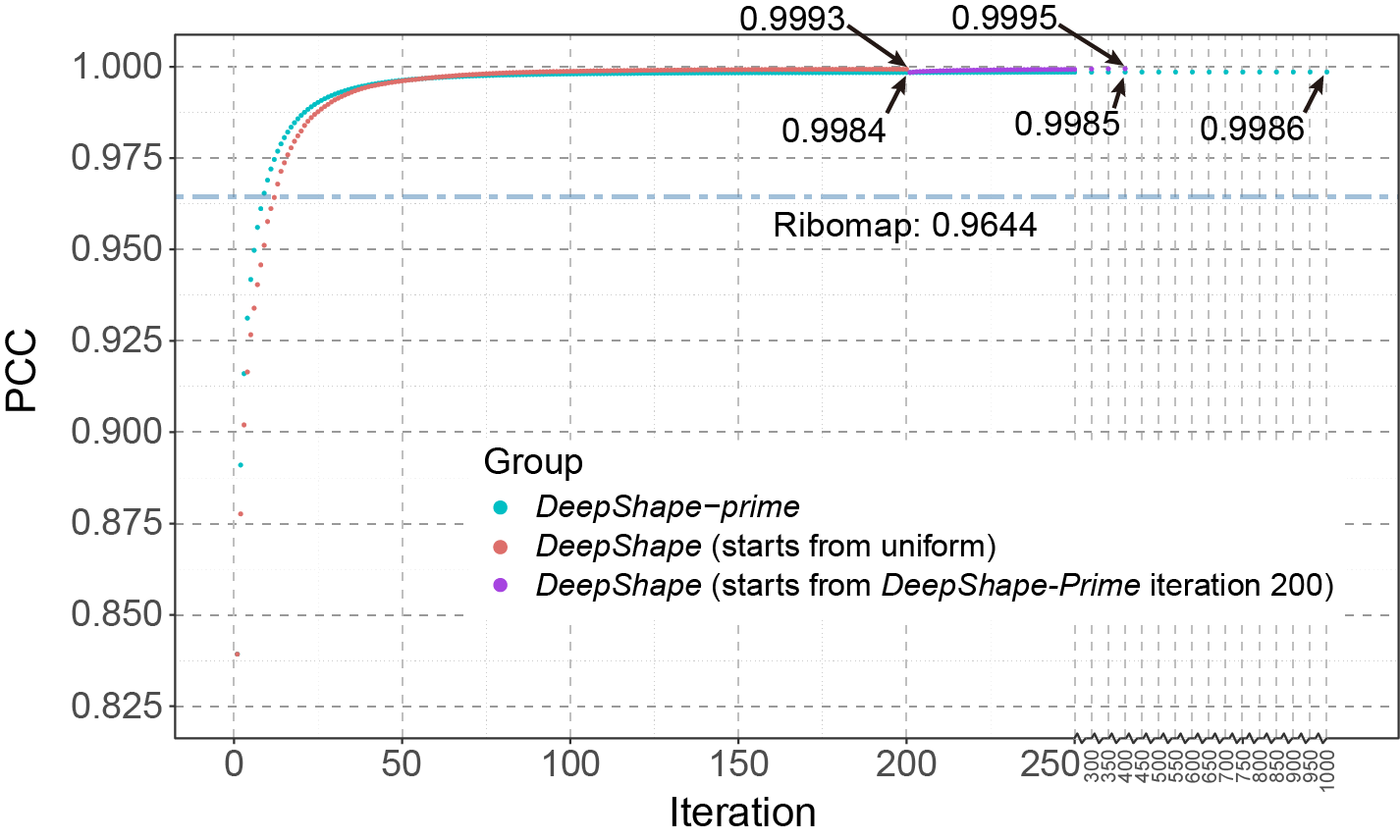


**Figure S3. PCC curves for prediction of length-normalized ribosome abundance at each iteration of *DeepShape* and *DeepShape-prime*.** For comparison, the PCC value of Ribomap is shown.

**
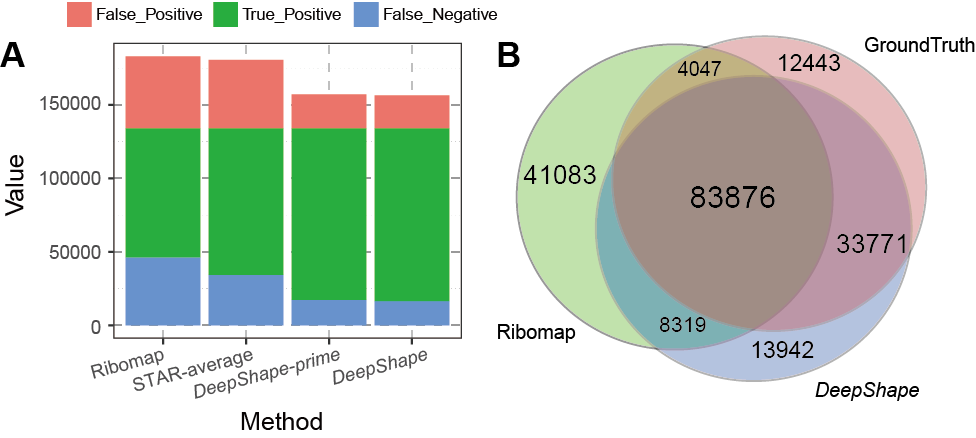
**

**Figure S4. Application of *DeepShape* to annotating ribosome stalling events.** **A.** False positive (red), true positive (green) and false negative (blue) when defining ribosome stalling events by Ribomap, STAR-uniform, *DeepShape-prime* and *DeepShape* on synthetic data. **B.** Venn diagram of ground truth, and stalling events found by *DeepShape* and Ribomap.

**
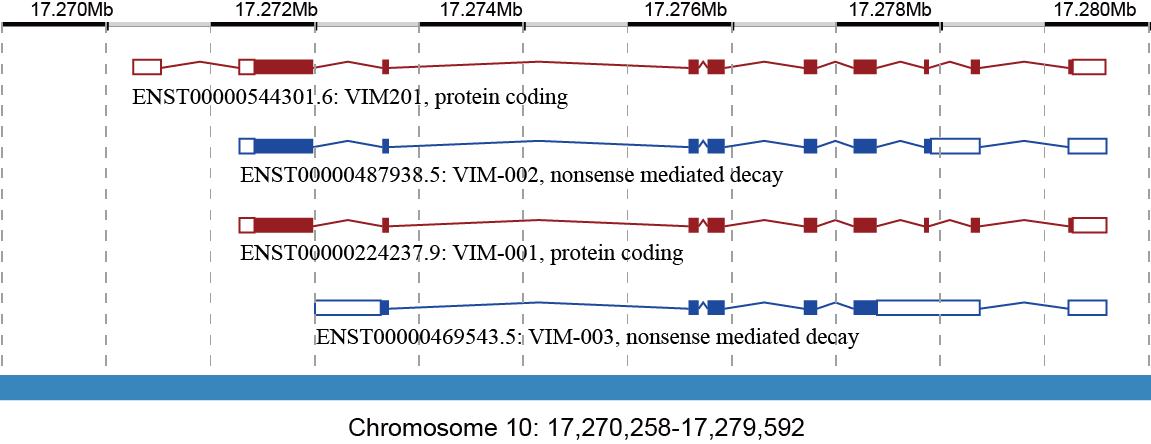
**

**Figure S5**. Four isoforms of the *VIM* gene.


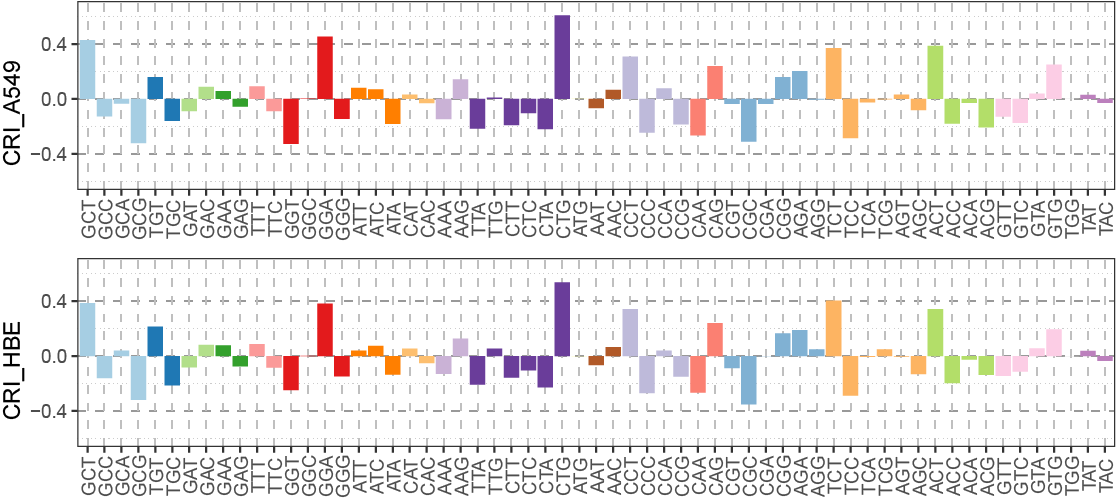


**Figure S6.** Codon Residence Index (CRI) of different codons in A549 and HBE cell. The data are from the work of Lian et al. (Lian, et al., 2016).


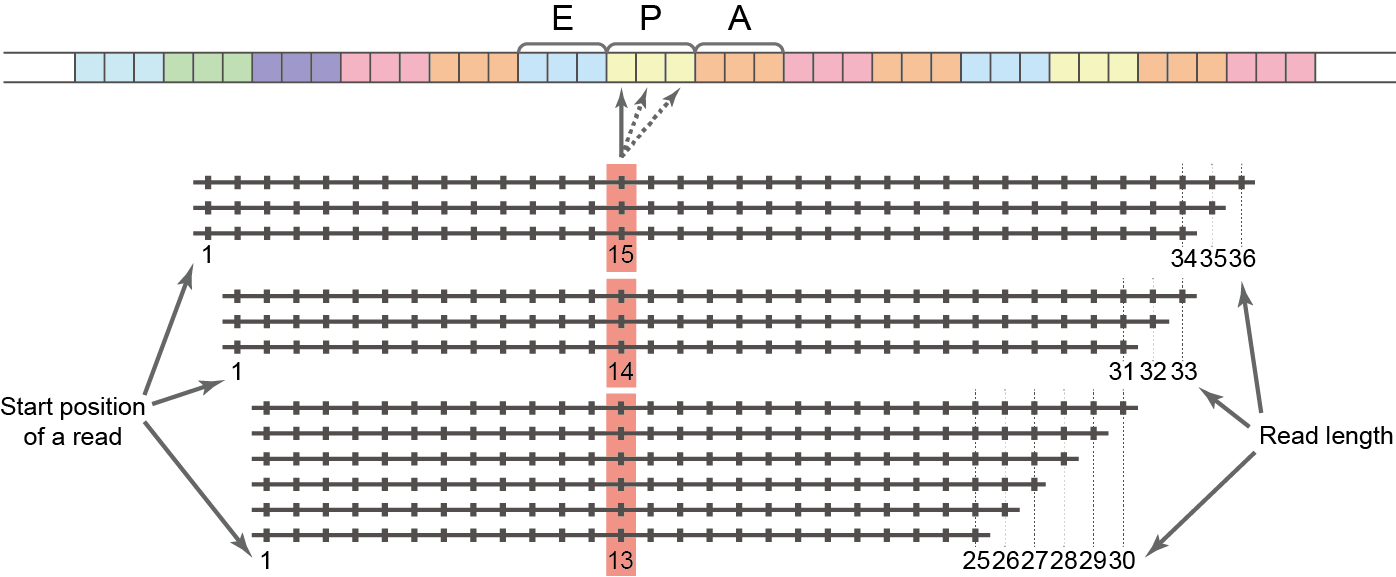


**Figure S7. Diagrammatic explanation of finding P-sites.** The 13th nucleotide for read length between 25bp and 30bp, 14th nucleotide for read length between 31bp and 33bp, and 15th nucleotide for read length between 34bp and 36bp was found as “P-site mark”. For most situations, the “P-site mark” would hit the first nucleotide of a codon, yet there still exists a portion of “P-site marks” hit the second and third nucleotide (Calviello and Ohler, 2017). In this study, if the “P-site mark” of an RPF read hit any position of a codon, this codon was then considered as the P-site of the ribosome.

**Table S1. Performance of shape model on genes with different lengths in *E. coli*.**

| **Length**  **(codons)** | **E.coli** | | **Mouse** | | **Human** | |
| --- | --- | --- | --- | --- | --- | --- |
|  | **RiboShape**  **V7** | **Shape model**  ***(DeepShape)*** | **RiboShape**  **V7** | **Shape model**  ***(DeepShape)*** | **RiboShape**  **V7** | **Shape model**  ***(DeepShape)*** |
| **<=250** | 0.49 | 0.54 | 0.33 | 0.35 | 0.33 | 0.38 |
| **251-500** | 0.52 | 0.54 | 0.36 | 0.37 | 0.37 | 0.4 |
| **501-750** | 0.51 | 0.54 | 0.38 | 0.38 | 0.30 | 0.41 |
| **751-1000** | 0.50 | 0.53 | 0.35 | 0.35 | 0.42 | 0.52 |
| **1001-1500** | 0.48 | 0.53 | 0.34 | 0.36 | 0.36 | 0.35 |
| **>=1501** | 0.49 | 0.54 | 0.34 | 0.29 | 0.31 | 0.34 |

**Table S2. TE and TE fold changes after PP242 treatment in the four cell invasion/metastasis genes**

| **Gene** | **Transcript** | **TE** | | | | **TE fold change** | |
| --- | --- | --- | --- | --- | --- | --- | --- |
|  |  | control 1 | control 2 | treat 1 | treat 2 | replicate 1 | replicate 2 |
| ***VIM*** | ENST00000544301.6 (VIM-001) | 1.21 | 1.33 | 0.35 | 0.31 | 0.29 | 0.23 |
|  | ENST00000469543.5 (VIM-003) | 21.95 | 1.42 | 7.7 | 0.01 | 0.35 | 6.97E-03 |
|  | ENST00000487938.5 (VIM-002) | 2.78 | 0.27 | 0.54 | 1.60E-21 | 0.2 | 6.00E-21 |
| ***YB1*** | ENST00000321358.11 (YBX1-201) | 0.69 | 0.69 | 0.23 | 0.16 | 0.33 | 0.23 |
| ***MTA1*** | ENST00000331320.11 (MTA1-001) | 0.41 | (DIV by 0) | 1.31E-03 | 1.78E-14 | 3.22E-03 | (DIV by NA) |
|  | ENST00000405646.5 (MTA1-003) | 0.34 | 0.47 | 0.43 | 0.22 | 1.29 | 0.47 |
|  | ENST00000438610.5 (MTA1-004) | 0.6 | 0.68 | 0.56 | 0.45 | 0.93 | 0.67 |
| ***CD44*** | ENST00000263398.10 (CD44-003) | 3.55 | 4.19 | 2.42 | 2.38 | 0.68 | 0.57 |
|  | ENST00000434472.6 (CD44-008) | 2.55 | 2.59 | 0.84 | 1.34 | 0.33 | 0.52 |

**Table S3. mRNA abundance, ribosome abundance and TEs in the four cell invasion/metastasis genes of A549 and H1299 cells**

| **Gene** | **Transcript** | mRNA abundance | | Ribosome abundance | | TE | |
| --- | --- | --- | --- | --- | --- | --- | --- |
|  |  | A549 | H1299 | A549 | H1299 | A549 | H1299 |
| VIM | ENST00000544301.6 (VIM-001) | 1194.8434 | 3124.08057 | 531.9457 | 3371.48 | 0.445201 | 1.079191 |
|  | ENST00000487938.5 (VIM-002) | 14.1875 | 7.69962 | 1.77E-16 | 10.70931 | 1.25E-17 | 1.390889 |
|  | ENST00000469543.5 (VIM-003) | 19.2018 | 6.12266 | 73.63336 | 799.0577 | 3.834711 | 130.5083 |
| YBX1 | ENST00000321358.11 (YBX1-201) | 847.094 | 1283.09 | 217.0944 | 231.1073 | 0.256281 | 0.180118 |
| MTA1 | ENST00000331320.11 (MTA1-001) | 0 | 9.46E-07 | 0.068562 | 0.001402 | - | - |
|  | ENST00000405646.5 (MTA1-003) | 26.6893 | 106.995 | 13.0905 | 12.72434 | 0.490478 | 0.118925 |
|  | ENST00000438610.5 (MTA1-004) | 8.87611 | 11.1743 | 0.001068 | 20.98162 | 0.00012 | 1.877667 |
| CD44 | ENST00000263398.10 (CD44-003) | 121.526 | 60.9086 | 168.4786 | 153.0632 | 1.386359 | 2.512998 |
|  | ENST00000434472.6 (CD44-008) | 25.725 | 4.67258 | 1.48E-17 | 9.342522 | 5.75E-19 | 1.999435 |

**References**

Calviello, L. and Ohler, U. Beyond Read-Counts: Ribo-seq Data Analysis to Understand the Functions of the Transcriptome. *Trends Genet* 2017;33(10):728-744.

Dobin, A.*, et al.* STAR: ultrafast universal RNA-seq aligner. *Bioinformatics* 2013;29(1):15-21.

Lian, X.*, et al.* Genome-Wide and Experimental Resolution of Relative Translation Elongation Speed at Individual Gene Level in Human Cells. *Plos Genet* 2016;12(2):e1005901.

Liu, T.Y. and Song, Y.S. Prediction of ribosome footprint profile shapes from transcript sequences. *Bioinformatics* 2016;32(12):i183-i191.

Patro, R.*, et al.* Salmon provides fast and bias-aware quantification of transcript expression. *Nat Methods* 2017;14(4):417-419.

Wang, H., McManus, J. and Kingsford, C. Isoform-level ribosome occupancy estimation guided by transcript abundance with Ribomap. *Bioinformatics* 2016;32(12):1880-1882.
